# A robust fuzzy rule based integrative feature selection strategy for gene expression data in TCGA

**DOI:** 10.1186/s12920-018-0451-x

**Published:** 2019-01-31

**Authors:** Shicai Fan, Jianxiong Tang, Qi Tian, Chunguo Wu

**Affiliations:** 1 School of Automation Engineering, University of Electronic Science and Technology of China, Chengdu, 611731 Sichuan China; 2 Center for Informational Biology, University of Electronic Science and Technology of China, Chengdu, 611731 Sichuan China; 3 Key Laboratory of Symbolic Computation and Knowledge Engineering of Ministry of Education, Jilin University, Changchun, 130012 China

**Keywords:** Integrative strategy, Expanded methylation data, Biomarker based feature selection, Robustness, Fuzzy rule, TCGA data

## Abstract

**Background:**

Lots of researches have been conducted in the selection of gene signatures that could distinguish the cancer patients from the normal. However, it is still an open question on how to extract the robust gene features.

**Methods:**

In this work, a gene signature selection strategy for TCGA data was proposed by integrating the gene expression data, the methylation data and the prior knowledge about cancer biomarkers. Different from the traditional integration method, the expanded 450 K methylation data were applied instead of the original 450 K array data, and the reported biomarkers were weighted in the feature selection. Fuzzy rule based classification method and cross validation strategy were applied in the model construction for performance evaluation.

**Results:**

Our selected gene features showed prediction accuracy close to 100% in the cross validation with fuzzy rule based classification model on 6 cancers from TCGA. The cross validation performance of our proposed model is similar to other integrative models or RNA-seq only model, while the prediction performance on independent data is obviously better than other 5 models. The gene signatures extracted with our fuzzy rule based integrative feature selection strategy were more robust, and had the potential to get better prediction results.

**Conclusion:**

The results indicated that the integration of expanded methylation data would cover more genes, and had greater capacity to retrieve the signature genes compared with the original 450 K methylation data. Also, the integration of the reported biomarkers was a promising way to improve the performance. PTCHD3 gene was selected as a discriminating gene in 3 out of the 6 cancers, which suggested that it might play important role in the cancer risk and would be worthy for the intensive investigation.

## Background

Biomarker based cancer diagnosis is a quite attractive and promising direction to improve the early cancer detection [1–3]. As its primary step, the investigation of the most discriminating genes between tumor and normal samples has been intensively carried out for more than two decades [4–8]. Generally the dataset has dozens or at most several hundred samples and millions or even more features for each sample, and it would cause the over-fitting problem that the selected optimized subsets are unstable [9, 10], or there would be many equivalent subsets with similar discriminating ability [11]. Till now, how to get the most robust combination is still an open question.

The integrative analysis based on gene expression and DNA methylation data had the potential to derive more reliable and robust gene signatures [12–17], and the fuzzy logic method has been suggested as an efficient way to incorporate biological knowledge with multi-omics data to built classification model [18]. However, integrative analysis is complicated by having a partial overlap because not all molecular levels are measured for all patients [14]. For the Cancer Genome Atlas (TCGA, https://portal.gdc.cancer.gov//) which provided multiple-omics data, the integration of gene expression and DNA methylation profiles could improve the molecular subtype classification [15]. As the 450 K methylation data only cover less than 2% of human genome, the integration with DNA methylation profile in a larger scale would expect more promising results.

In this work, a more robust gene signature selection strategy was developed by integrating gene expression data, expanded DNA methylation data and prior knowledge. The strategy mainly include two steps: firstly, the integrative analysis was implemented on the RNA-seq data and expanded DNA methylation profile, the methylation profile was retrieved from a newly developed expanding algorithm [19], and included ~ 18 times more CpG sites than 450 K methylation array data; then, the candidate gene features were further selected based on its combination performance with the reported biomarkers.

Fuzzy rule based classification method was applied in the model construction for its easy understanding of the results [20]. On 6 cancer data from TCGA (BRCA, PRAD, LIHC, HNSC, KIRP and THCA), the prediction performances of these selected genes in the 10-fold cross validation were close to 100%, indicating that our selected gene features could classify the tumor and normal samples quite well. Applying other 4 gene feature selection models on the TCGA data, the cross validation results of three models were quite similar to our results. However, our proposed strategy demonstrated obvious better prediction performance on independent test data, indicating that gene signatures selected with our strategy were more discriminative to distinguish tumor samples from the the normal, and therefore, was more robust.

The fuzzy rules derived from the selected genes could provide the gene expression patterns of different cancers, which would be meaningful to understand the discriminant function explicitly. The discriminating genes that most commonly shared among the 6 cancers were CDKN2A and PTCHD3, which were both selected in three cancers. CDKN2A has been widely reported to act as a potential biomarker [21, 22], while there were few reports about the cancer risk of abnormal expression of PTCHD3, which definitely shed light on the intensive investigation of PTCHD3 for its role in cancers.

## Methods

### Strategy of the robust gene feature selection

**Fig. 1:**
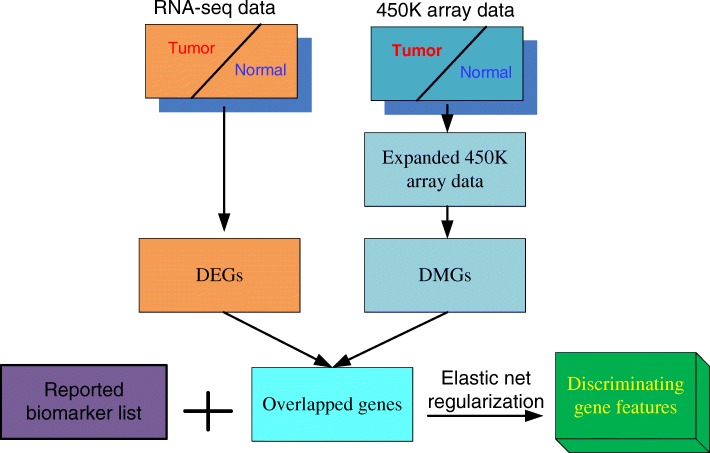
The strategy of the integrative gene feature selection

Aim to get the more robust gene signatures in tumors, an integrative selection strategy was proposed in this work. As shown in Fig. 1, the genes that were simultaneously differentially expressed and methylated were firstly selected as the candidate feature genes. Then, to make full use of the previous research results, the reported biomarker genes for each cancer were weighted in elastic-net regularized generalized linear models (glmnet package in R) to find the best combination genes from these candidate feature genes.

When considering the methylation data, we applied the expanding algorithm [19] to get the expanded methylation profile as the methylation landscape from 450 K methylation array is quite limited. The expanding algorithm predicted the methylation value of CpG site based on its most neighboring 450 K probes and its local methylation pattern, and illustrated high prediction accuracy. The expanded methylation profile was with 18 times more methylation loci than the original 450 K array.

When calculating the Differentially Methylated Genes (DMGs) between tumor and normal samples, the Differentially Methylated Loci (DML) were firstly calculated. A DML was a CpG locus which satisfied two requirements, firstly, it showed significantly higher/lower methylation value among tumor samples compared with normal samples in t-test (the adjusted *p*-value < 0.01); secondly, its absolute differential methylation value between tumor and normal samples was larger than 0.1. Only the genes whose promoter regions (TSS-1500,TSS + 500) contained any DML were considered. Then the Fisher’s combined test was used to get the q-value to evaluate whether a gene is differentially methylated.

For the RNA-seq expression data of the 6 cancers, we firstly transformed them with log2 function, and then compared the expression level of each gene between tumor and normal samples with t-test. And only the significantly Differentially Expressed Genes (DEGs) were selected (the criteria were with absolute fold change > 1, and adjusted *p*-value < 0.01).

### Model construction

**Fig. 2:**
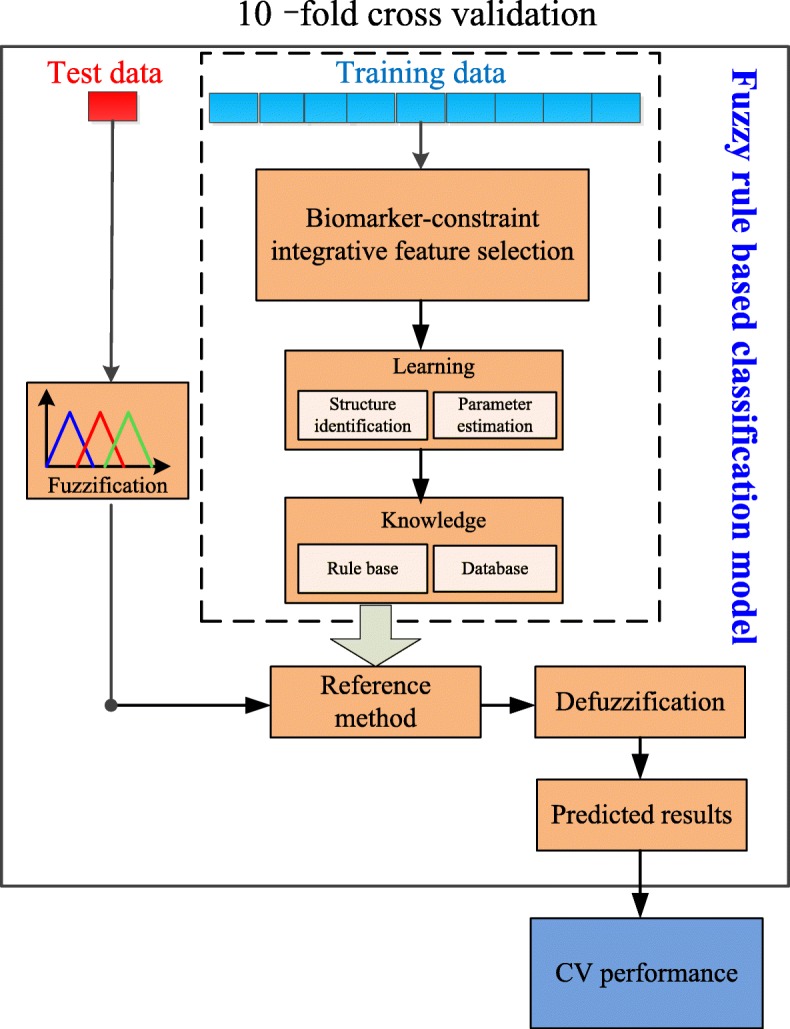
The pipeline of our fuzzy rule based classification model

Rule based classification model was popularly applied in the classification to understand how the gene features could affect the prediction. Fuzzy rule based classification is an extension of the classical set theory to model sets whose elements have degrees of membership [20]. Our classification model was constructed with cross validation strategy and the pipeline was shown in Fig. 2.

With the selected gene features, the input spaces of the gene expression data were normalized to the range of [0, 1], and divided into 3 linguistic terms with “small”, “medium” and “large”. We chose the membership function to be Gaussian function, and the distribution parameters were *N*(0, 0.175), *N*(0.5, 0.175)and *N*(1, 0.175). Then the fuzzy IF-THEN inferences based on the database were generated using the Mamdani model, and this process was repeated for all samples in the training data, and the final IF-THEN rules were output.

Similarly, the gene features selected based on only reported biomarkers, RNA-seq data, or gene features selected based on the integrative features of RNA-seq data and the original 450 K methylation data, or the integrative gene features that could be best combined with biomarkers, were also used to build the corresponding fuzzy rule based models.

The performances of all the fuzzy rule based models were evaluated with cross validation strategy. The sample data of each cancer were divided into 10 folds, 9 folds were used to train the classification model, and the remaining fold was applied to test the performance of the training model, and the operation was repeated for 10 times.

### Datasets

Cancer data were retrieved from TCGA, and RNA-seq data and 450 K methylation data of 6 cancers (BRCA, PRAD, LIHC, HNSC, KIRP, THCA) were selected because of their reasonable sample size.

The potential biomarkers for each cancer were derived from literature reports, the numbers of biomarkers used in the work for BRCA, PRAD, LIHC, HNSC, KIRP and THCA were 9, 6, 8, 7, 8 and 7, respectively, and were listed in Table 1.

**Table 1:** The list of selected biomarkers for each cancer

Cancer Type	Applied biomarker	Reference
BRCA	ESR1, ERBB2, MKI67, CCND1, CCNE1, ESR2, BRCA1, BRCA2, PGR	[27, 28]
PRAD	PCA3, PTEN, AMACR, KLK3, MALAT1, GOLM1	[1, 29]
LIHC	AFP, DKK1, VEGFA, IGF1, IL6, CXCR2, CCR2, EP400	[30]
HNSC	CCR7, CD44, CEP55, CTTN, CXCR4, MMP2, NFKB1	[31]
KIRP	VHL, STC2, VCAN, VEGFA, CA9, VCAM1, HIF1A, BIRC5	[32, 33]
THCA	LGALS3, MET, BRAF, RET, HRAS, PAX8, PPARG	[34]

For the performance comparisons, the independent gene expression data for the 6 cancers were retrieved from Expression Project for Oncology (expO, ftp://ftp.ncbi.nlm.nih.gov/pub/geo/DATA/SeriesMatrix/GSE2109/). And the gene expression data were normalized to the RNA-seq expression levels based upon quantiles to reduce the batch effect.

## Results

### Summary of the discriminating gene features

Based on the strategy of gene feature selection, the number of DEGs from RNA-seq data and DMGs from the expanded methylation data for BRCA, PRAD, LIHC, HNSC, KIRP and THCA were listed in Table 2.

**Table 2:** The numbers of DEGs, EDMGs and DMGs

Cancer Type	#DEGs	#DMGs from the expanded methylation data	#DMGs from the original 450 K array
BRCA	1702	2480	2275
PRAD	901	2516	2233
LIHC	1665	3315	3009
HNSC	1417	2845	2700
KIRP	2192	1738	1521
THCA	1319	1053	569

Compared with DMGs from the original 450 K methylation array data (see Table 2), one could see that the expanded methylation landscape could provide more DMGs. The increased numbers were 205, 283, 306, 145, 217 and 484.

Then after extracting the most informative gene features combined with reported biomarkers using elastic net regression model, the most discriminate feature genes were finally obtained. The selected gene numbers for BRCA, PRAD, LIHC, HNSC, KIRP and THCA were 47, 23, 50, 21, 30 and 12, respectively.

### Performance description and comparisons

**Fig. 3:**
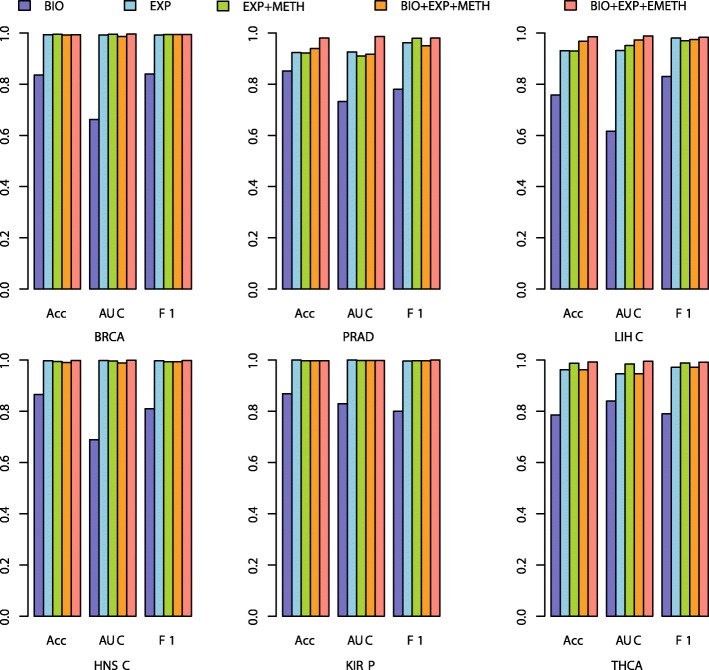
The cross validation results on 6 cancers from TCGA with different feature selection models

Based on these selected discriminating gene features, the prediction results using fuzzy rule based classification model in 10-fold cross validation were shown in Fig. 3 (BIOmarker+EXPression+Expanded METHylation, BIO + EXP + EMETH). The prediction accuracies and AUCs were all close to 100% in all of the 6 cancers.

Also we observed the cross validation performances of other four models with elastic-net regularized generalized linear model. They are: 1) Model based on only reported biomarkers (BIO); 2) model based on gene features selected from only RNA-seq data (EXP); 3) model based on gene features selected based on the integrative features of RNA-seq data and the original 450 K methylation array data (EXP + METH); and 4) the gene features from model EXP + METH were further selected based on its combination performance with the reported biomarkers (BIO + EXP + METH). Their cross validation results were also shown in Fig. 3. One could see that the cross validation performance of BIO model (the Acc and AUC were both around 0.8 in all of the 6 cancers) was relatively worse than other models. The cross validation performances of the other three models were similar to our BIO + EXP + EMETH model, and were all close to 100%, but the numbers of selected gene features and the gene lists in the four models were different. This is consistent with the previous results that there would be multiple equivalent gene combinations that showed discriminating ability.

**Fig. 4:**
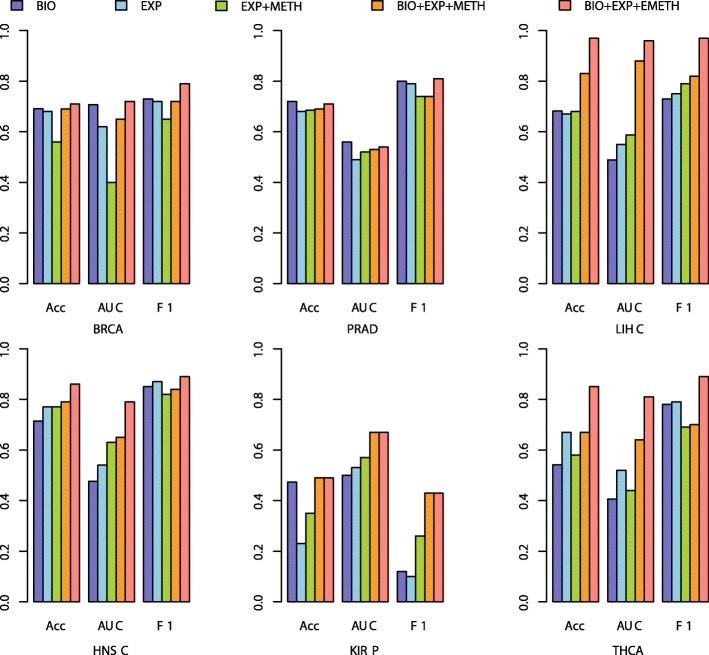
The prediction results on independent datasets with different feature selection models

To evaluate the reliability of these gene feature selection strategies above, some independent gene expression data of these cancers were extracted and predicted with the trained models. The prediction performances of each model on the 6 cancers were shown in Fig. 4. One could see that the performances of the 5 models mentioned above varied. Our proposed BIO + EXP + EMETH model always outperformed other models in all of the 6 cancers, indicating that our strategy could select the more robust gene signatures between tumor and normal samples. The performances of BIO + EXP + METH model were worse than the BIO + EXP + EMETH model, indicating the superior performance when integrating with the expanded 450 K methylation data compared with the original 450 K methylation data. The result that BIO + EXP + METH model was better than EXP + METH model indicated that the reservation of the reported biomarkers could improve the prediction precision.

It is reasonable because what we emphasized in our strategy had obvious advantages over other models, firstly, the reservation of the reported biomarkers was a way to make use of the previous knowledge in cancer diagnosis; secondly, the expanded 450 K methylation data could introduce some important DMGs which missed in the original 450 K array data.

### Fuzzy rules and gene analysis

Another feature of our strategy was the application of fuzzy rule based classification model, which was more robust than hard rule based classification model, and could output classification rules easily understood by biologists. Taking THCA as an example, 12 genes were finally selected in the model, and one of the corresponding fuzzy rule for thyroid carcinoma classification was: IF (APOD is small) ^ (CDKL2 is large) ^ (CLEC4F is medium) ^ (CSF2 is small) ^ (HAPLN1 is medium) ^ (ITIH2 is medium) ^ (KLHDC8A is large) ^ (KLK13 is medium) ^ (MMP23A is small) ^ (MYOC is small) ^ (R3HDML is small) ^ (RBP4 is medium), THEN the sample is a tumor sample.

Furthermore, we were interested in the selected gene features in each cancer (shown in Table 3), especially the genes simultaneously selected in several cancers. The top overlapped genes were CDKN2A (in BRCA, KIRP and LIHC) and PTCHD3 (in PRAD, HNSC and KIRP). CDKN2A is a gene acting as a tumor suppressor by regulating the cell cycle, therefore, it is reasonable that its abnormal expression pattern would be a common signature in different cancers, and it has been reported as a potential biomarker in previous reports [21, 22]. For PTCHD3, it is a gene generally expressed in germ cells of the testis [23], and what the most interesting observation is that there are only two reports about its possible correlation with colorectal cancer [24, 25]. As PTCHD3 indicated its discriminating ability in three cancers in our research, we speculate that it may be a potential biomarker for PRAD, HNSC and KIRP, and it is meaningful to carry out biological validation in the next step.

**Table 3:** The selected gene signatures in each of the cancer

Cancer Type	Selected gene signatures
BRCA	ABCB5, ADAMTS5, ALX4, ANPEP, APCDD1, ARHGAP20, BCHE, BRCA2, CCL11, CCL28, CDKN2A, CEBPA, CHRNA6, CNN1, COL6A6, CX3CL1, DNAH14, EMILIN2, ERBB2, FGF10, GRID1, GSN, HTR2A, KCNJ2, KCNMB1, KLB, KRT4, LEP, LOXL1, LRIT2, MYOC, NRN1, OLFM3, OR10A3, OXTR, P4HA3, PENK, PRSS55, PSG3, RAX2, RPE65, SH3TC2, TBX15, TPO, VGLL2, WISP1, WT1
PRAD	AMDHD1, AMY2B, AQP5, C17orf102, C19orf45, CHST4, GABRR1, GCKR, HSD17B3, IL17A, LTK, OVCH2, PTCHD3, PTGS2, RPL10L, SEPT12, SOX8, TRH, TYR, UGT2B10, UGT3A1, VSIG10L, ZNHIT2
LIHC	ADCYAP1R1, AMPD1, ANKRD34A, ANKRD34C, B3GALT5, B4GALNT1, C1orf177, CASQ2, CCL19, CD207, CDH13, CDKN2A, CSPG4, DMC1, EBF2, ECM1, ELAVL2, EPO, FHL5, GJA1, GJC1, GLYATL2, GOLGA8A, GYPA, HBB, HBD, HSF4, HSPG2, IFITM4P, IL20RA, IL20RB, IRX3, LCE2D, MARCH4, MKRN3, NPAS4, NRXN1, OR51E2, PCSK2, PDZD2, PKMYT1, PMP2, RBM11, SEMA5B, SFTA1P, SLC17A8, SLCO1C1, STC2, TAC1, TM4SF18
HNSC	ALG1L, BOC, CA13, CLDN10, CMA1, CNTFR, DIXDC1, FGFR2, FOXS1, GBX2, GPT, HCN1, HOXC6, HOXC9, HOXD10, HOXD9, HPR, KALRN, KIR2DS4, LAIR2, LPPR5, MARCH4, MMP13, PAEP, PCDHGA9, PCDHGB7, PCK1, PHGDH, PIK3R1, PTCHD3, RIMS4, SCIN, SDPR, SLC46A2, SLC5A8, SORBS2, SOX11, SPP1, SRD5A2, SVIP, TAC1, TGFBR3, TMEM132C, TMEM217, TRPC4, ZIC5, ZNF132, ZNF43, ZNF486, ZNF608, ZNF626, ZNF677, ZNF813, ZNF844
KIRP	ABCA4, AFAP1L2, ALDOB, ASPG, B3GALT2, BIRC5, C17orf78, CA9, CCNI2, CDKN2A, CHP2, CLDN19, ENOX1, FOXD2, GINS2, GREM2, INSRR, KIFC2, KNG1, MET, MUC15, MYOM1, NOTUM, PLIN2, PTCHD3, SFRP1, SIAH3, SPRR2A, ST6GALNAC3, VEGFA
THCA	APOD, CDKL2, CLEC4F, CSF2, HAPLN1, ITIH2, KLHDC8A, KLK13, MMP23A, MYOC, R3HDML, RBP4

## Discussion

Due to the complex nature of cancer, the investigation of potential biomarkers for cancer diagnosis is still far from the end. And the current feature selection methods suffer from the lack of robustness.

Aim to improve the robustness of feature selection, a gene feature selection strategy was proposed in this work. The DEGs were firstly filtered with the requirement that they should be simultaneously differentially methylated. Because of the low coverage of 450 K methylation array, an expanding algorithm was applied to get 18 times more CpG loci, and produced more DMGs. Then these candidate gene features were further screened based on their combination performance with some reported biomarkers.

Besides the high cross validation performance on TCGA data, our proposed gene feature selection strategy could get better prediction performances on six independent cancer gene expression data, which verified our conclusion that the proposed feature selection strategy would be more robust. And the PTCHD3 gene, which indicated discriminating ability in cancer prediction in three cancers, was worth for further exploring.

From the prediction results on the independent data (Fig. 4), the performances based on the EXP + METH were better than the EXP model in 4 of the 6 cancers, while the performances in BRCA and THCA were worse, which was consistent with some concerns that the simple integration of gene expression data and 450 K methylation data might not achieve better prediction results [26]. Therefore, the integrative analysis with expanded 450 K array data would be a promising direction for better performance. For the prediction results on KIRP, one could see that none of the models could get satisfying classification results. We speculate that the KIRP samples of TCGA and expO might be from quite different subtypes, and the discriminating patterns based on TCGA samples do not reflect the abnormal patterns in tumor samples from expO.

In our strategy, the expanded 450 K methylation data were retrieved based on an expanding algorithm, whose prediction accuracy is around 90%. It is the truth that the methylation values of some expanded CpG loci may not reflect their true methylation status, but our method could reduce its affection. Firstly, the definition of DMG was based on all the promoter CpG loci included in the expanded profile, the inaccuracy of one locus or small proportion of promoter CpG loci would not obviously affect the methylation status of the whole promoter; secondly, the candidate gene features should be not only differentially methylated, but also differentially expressed, therefore, the filtering requirements also helped filter the false differentially methylated genes caused by the inaccurate prediction of the expanding algorithm.

## Conclusions

In this paper, we proposed a novel multi-omics data integration strategy to retrieve the robust gene signatures in the classification of tumor and normal samples, and the proposed strategy could achieve the best prediction performances comparing with other models. The strategy could be applied in the integrative analysis of other omics data, especially the idea of the application of expanding the limited 450 K methylation array data.

## Abbreviations

BIO + EXP + EMETH: BIOmarker+EXPression+Expanded METHylation
BIO + EXP + METH: BIOmarker+EXPression+METHylation
BIO: BIOmarkers
BRCA: Breast invasive carcinoma
DEG: Differentially Expressed Genes
DMGs: Differentially Methylated Genes
DML: Differentially Methylated Loci
EXP + METH: EXPression+METHylation
EXP: EXPression
HNSC: Head and neck squamous cell carcinoma
KIRP: Kidney renal papillary cell carcinoma
LIHC: Liver hepotocellular carcinoma
PRAD: Prostate adenocarcinoma
TCGA: the Cancer Genome Atlas
THCA: Thyroid carcinoma
